# Authors’ response: Plenty of coronaviruses but no SARS-CoV-2

**DOI:** 10.2807/1560-7917.ES.2020.25.8.2000197

**Published:** 2020-02-27

**Authors:** Chantal B Reusken, Bart Haagmans, Adam Meijer, Victor M Corman, Anna Papa, Remi Charrel, Christian Drosten, Marion Koopmans

**Affiliations:** 1 Centre for Infectious Disease Control, National Institute for Public Health and the Environment, Bilthoven, the Netherlands; 2 Viroscience department, Erasmus MC, Rotterdam, the Netherlands; 3 Charité - Universitätsmedizin Berlin Institute of Virology, Berlin, Germany and German Centre for Infection Research (DZIF), Berlin, Germany; 4 Department of Microbiology, Medical School, Aristotle University of Thessaloniki, Thessaloniki, Greece; 5 Unité des Virus Emergents (Aix-Marseille Univ-IRD 190-Inserm 1207-IHU Méditerranée Infection), Marseille, France.

**Keywords:** Coronavirus, respiratory infections, SARS-CoV-2

**To the editor: **The emergence of a novel pathogen raises a wide range of urgent questions that need to be addressed to guide clinical and public health responses [1]. One of the cornerstones and a prerequisite for a proper public health and clinical response is the availability of a reliable diagnostic and reference laboratory service with adequate capacity. This is recognised in the International Health Regulations (IHR 2005) and explicitly recognised by the World Health Organization (WHO) and the European Centre for Disease Prevention and Control (ECDC) in their risk assessments and guidelines upon the emergence of severe acute respiratory syndrome coronavirus 2 (SARS-CoV-2) [2–5]. Colson et al. question the proportionality of the laboratory readiness and response for this novel coronavirus in expert laboratories of Emerging Viral Diseases-Expert Laboratory Network (EVD-LabNet) and European Reference Laboratory Network for Human Influenza (ERLI-Net) in 30 European Union and European Economic Area (EU/EEA) countries, as outlined in our publication [6]. The initial response to the emergence of SARS-CoV-2 is a strategy of containment consisting of active case finding in combination with case isolation and quarantine of contacts [3], a strategy for which adequate worldwide laboratory services are indispensable as seeding of the virus through travellers from China could be expected. Indeed, during the response period for our survey, the first cases of coronavirus disease 2019 (COVID-19) were already notified in France on 24 January and as at 26 February 2020, 381 cases have been notified in Europe [6,7].

While the current response strategy is still focused on containment, it is becoming increasingly clear that the epidemic may turn global, in which case mitigation will be the next option to control the impact of the pandemic. Substantial autochthonous circulation in Italy, Iran and South Korea has been reported by now, while there is increasing evidence that subclinical infections occur and people may be infectious before symptoms appear [8–10]. These developments indicate that the window of opportunity to contain and eradicate is rapidly narrowing. Colson et al. remark that “*the emergence of SARS-CoV-2 in December 2019 reproduced this pattern of disproportionate fear of importation and spread in mainland France while the cases reported worldwide remain almost only localised in China as only 34 people died of this disease (COVID-19) outside China*” thus may rapidly become invalid with the increasing instances of ongoing transmission in locations outside China. Already in the past 24 h (25 to 26 February 2020), nine new deaths outside China were reported [11]. Therefore, it is needless to state that for each phase in the control and mitigation of a novel emerging pathogen, adequate laboratory preparedness and response are crucial. The readiness survey that we performed served the important purpose of mapping the initial response capacities in Europe and identifying barriers to diagnostic implementation.

Therefore, we maintain that the urgent implementation and monitoring of diagnostic capacities and capabilities for SARS-CoV-2 in Europe is proportional to the containment and expected mitigation phase of the global public health response and is critical for care of local patients. The implementation of SARS-CoV-2 diagnostics does not replace nor exclude diagnostic capacity for other respiratory viruses. Moreover, it explicitly does not disregard the importance of other seasonal respiratory pathogens for public and patient health. This is clearly illustrated by the authors themselves, who indicate that they have tested thousands of samples of patients suspected of respiratory viral disease not only for common respiratory pathogens but also for SARS-CoV-2. The fact that Colson et al. found a wide range of seasonal respiratory viruses indeed underlines the impact of such pathogens. Furthermore, it clearly shows that diagnosis of respiratory viruses cannot be done syndrome-based and supports the need for the availability of diagnostics for a panel of pathogens including SARS-CoV-2.

The proportionality of the measures taken to control SARS-CoV-2 can only be rightly evaluated when the critical questions related to SARS-CoV-2, including the exact mortality rate highlighted by the authors, have been answered. These answers are already largely known for the common seasonal respiratory viruses but until then cautiousness, is needed. Regardless of the outcomes of the rapidly evolving epidemiological situation of SARS-CoV-2 and even in the event that SARS-CoV-2 becomes the fifth commonly circulating human coronavirus, the need for diagnostic capacity and capability will remain.
